# Case Report: Adolescent epididymal cysts: three management approaches including a rare synchronous epididymal cyst and testicular torsion

**DOI:** 10.3389/fsurg.2025.1660670

**Published:** 2025-11-11

**Authors:** Qichao Xu, Cancan You, Jianhong Wu, Qiongzhang Xia, Congde Chen, Liguang Xia

**Affiliations:** Department of Pediatric Urology, The Second Affiliated Hospital and Yuying Children’s Hospital of Wenzhou Medical University, Wenzhou, Zhejiang, China

**Keywords:** case report, epididymal cyst, scrotal emergency, torsion, spermatocele

## Abstract

**Introduction:**

Epididymal cysts are common benign lesions that typically present as painless scrotal masses. They predominantly occur in adult males but can also be found in adolescents, albeit with a higher likelihood of being overlooked at this age. Most patients experience relief or complete resolution of symptoms following conservative treatment. However, large cysts pose a risk of torsion. Torsion of an epididymal cyst in adolescents is clinically rare, with only a few reported cases to date, presenting diagnostic challenges for clinicians.

**Case descriptions:**

We report three cases of epididymal cysts in adolescents. Case 1 involved an asymptomatic cyst treated with aspiration, demonstrating significant cyst reduction at 4-month follow-up. Case 2 presented a persistent simple cyst managed by elective surgical excision with pathological confirmation after prolonged surveillance showed no resolution. Case 3 presented acutely with pain and suspected torsion on scrotal ultrasound, requiring emergency surgical intervention that revealed a 720° torsion of the epididymal cyst combined with 180° testicular torsion, treated testis detorsion and cyst excision. All cases achieved favorable outcomes.

**Conclusion:**

Epididymal cysts in adolescents warrant a tailored management strategy. While aspiration or elective excision are effective for non-acute cases, the potential for synchronous testicular torsion necessitates a high index of suspicion and prompt surgical exploration in acute presentations to preserve testicular viability.

## Introduction

An epididymal cyst is a benign cystic lesion that can develop anywhere along the epididymis. According to the literature, the incidence of epididymal cysts in pediatric populations ranges from 5% to 20% (1, 2). In post-pubertal adolescents, a cyst located specifically in the caput (head) that contains spermatozoa is termed a spermatocele; both entities, however, share similar clinical implications and management considerations. This condition typically lacks specific clinical symptoms, and most cases are incidentally detected during routine testicular ultrasound examinations (3). A minority of patients present with a palpable scrotal mass, which may exhibit positive translucency on examination, often leading to confusion with hydrocele. Larger cysts pose a risk of torsion (4), and patients experiencing ipsilateral scrotal pain may be misdiagnosed with conditions such as epididymitis, orchitis, or other scrotal disorders, potentially delaying appropriate treatment (5). Therefore, accurate diagnosis is critical for optimal patient outcomes. This study retrospectively analyzed three cases of adolescent epididymal cysts treated at our institution and reviewed relevant literature. This review aims to summarize the clinical features and management strategies for epididymal cysts in adolescents, thereby enhancing pediatric surgeons’ understanding of this condition, reducing diagnostic errors and complications, and guiding personalized treatment approaches for affected children.

## Case descriptions

### Case 1

**Figure 1:**
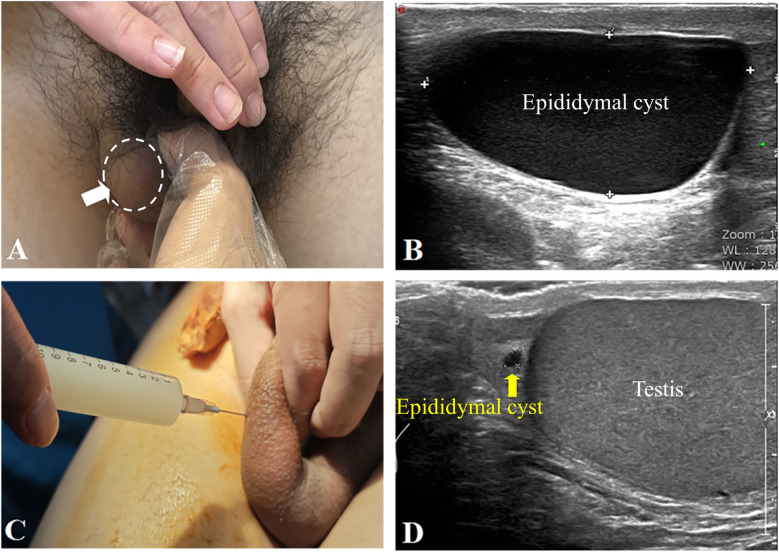
**(A)** A cystic mass was palpated in the right scrotum (indicated by a white arrow), measuring approximately 3 × 4 cm. **(B)** Scrotal ultrasound imaging revealed marked enlargement of the right epididymal head, with a hypoechoic area measuring 40 × 22 × 34 mm(epididymal cyst). The epididymal cyst had clear boundaries, poor internal sound transmission, and demonstrated a posterior acoustic enhancement. **(C)** During the procedure, approximately 20 ml of milky, opaque fluid was aspirated from the right scrotal mass. **(D)** Four months post-surgery, follow-up scrotal ultrasound confirmed a significant reduction in the size of the right epididymal head cyst to 3 × 2 × 2 mm (Figure 1D).

A 15-year-old adolescent, previously healthy, presented to our hospital for evaluation and management of phimosis due to a small foreskin opening that had persisted for one year. On physical examination, both testicles were in normal position. The foreskin opening was noted to be narrow and non-retractable. A cystic mass, approximately 3 × 4 cm in size, smooth, non-adherent, and non-tender, was palpated in the right scrotum (Figure 1A). The transillumination test was positive, while no abnormalities were detected in the left scrotum. Scrotal ultrasound examination revealed “prominent enlargement of the head of the right epididymis with a well-defined anechoic area measuring 40 × 22 × 34 mm, poor internal acoustic transmission, and posterior acoustic enhancement” (Figure 1B), suggestive of Epididymal cyst. After explaining the condition to the family, surgical excision of the this cyst was recommended. The family, however, declined surgical excision due to concerns over invasiveness and potential long-term impacts on testicular function. Following a shared decision-making process where all options—including observation, aspiration (with its high recurrence risk), sclerotherapy (with its uncertain risk of epididymal damage), and surgical excision—were discussed, we opted for diagnostic cyst aspiration during the planned circumcision to obtain fluid for analysis. All procedures were performed in an outpatient operating room with the patient in the supine position. After routine disinfection of the penis and scrotum with 0.5% povidone-iodine, the planned puncture site over the right scrotal cyst was anesthetized by local infiltration with 1% lidocaine. Approximately 20 mL of milky, opaque fluid was successfully aspirated using a disposable 10 mL syringe (Figure 1C). Following this, the surgical field was re-disinfected, and a circumcision was performed. The procedure was well-tolerated with stable vital signs and no reported pain. The patient was monitored in the recovery room for 30 min with no immediate complications (e.g., bleeding, hematoma) noted. Given that the appearance of the aspirated fluid is pivotal for differentiating a spermatocele from an infectious collection, the sample was sent for laboratory analysis, including semen analysis and microbiological culture, to establish a definitive diagnosis. Semen analysis revealed a high concentration of sperm. Bacterial culture results for Neisseria gonorrhoeae were negative. Based on the patient's medical history, physical examination, and auxiliary examinations, the final diagnosis was a spermatocele. Four months post-procedure, the child returned for follow-up. He reported no symptoms related to the right scrotum. Physical examination showed no significant differences between the two testes, and no obvious mass was palpable in the right scrotum. Follow-up scrotal ultrasound confirmed a significant reduction in the size of the right epididymal head cyst to 3 × 2 × 2 mm (Figure 1D).

### Case 2

**Figure 2:**
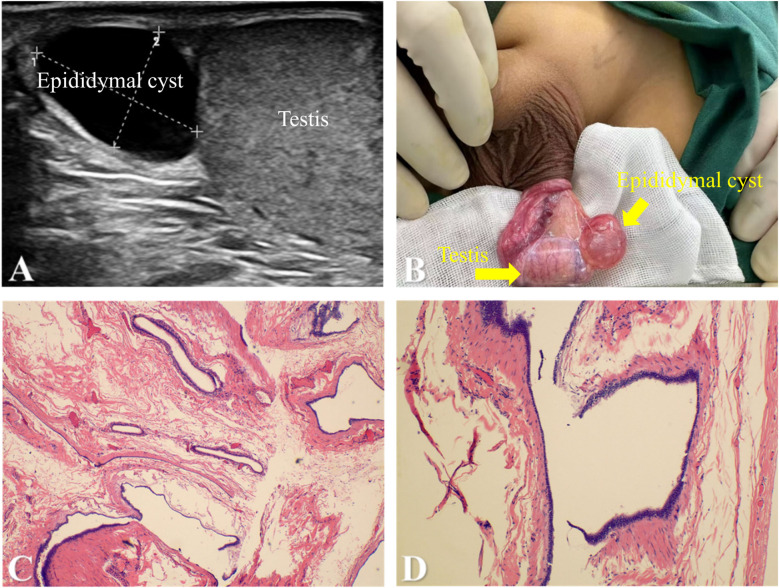
**(A)** scrotal ultrasound revealed a hypoechoic fluid-filled area above the left testis within the scrotum, measuring approximately 17 × 12 × 17 mm. The mass(epididymal cyst) had clear boundaries and exhibited good sound transmission. **(B)** Intraoperative findings revealed a mass superior to the testis, which was identified as enlarged epididymal tissue measuring approximately 20 × 17 × 15 mm. **(C)** and **(D)** Microscopic examination revealed cystic wall tissue lined by a single layer of cuboidal to flattened epithelium with focal ciliation. The cyst wall consisted of fibrous connective tissue with scattered bundles of smooth muscle.

A 12-year-old adolescent patient presented to our hospital for surgical evaluation due to a left scrotal mass that had been present for one year. One year ago, the patient was noted to have a left scrotal mass without associated scrotal erythema or pain. He sought medical attention at a local hospital, where an ultrasound revealed a left epididymal cyst measuring approximately 19 × 17 × 16 mm. Over a one-year outpatient follow-up, the cyst size remained stable, measuring approximately 20 × 18 × 16 mm six months ago and 19 × 17 × 17 mm three months ago. The family now requests surgical intervention. The patient had no prior history of scrotal trauma. On physical examination, a cystic mass measuring approximately 2 × 2 cm was palpated in the left scrotum. The mass was smooth, non-tender, and non-adherent, with a positive transillumination test. No abnormalities were noted in the right scrotum. A scrotal ultrasound confirmed “a well-defined anechoic area measuring 17 × 12 × 17 mm above the left testis” (Figure 2A). A diagnosis of the left epididymal cyst was made. Given the lack of resolution over the prolonged follow-up period and the family's strong preference for surgery, the patient underwent resection of the left epididymal cyst under general anesthesia. Intraoperatively, the cyst was located at the head of the epididymis and measured approximately 20 × 17 × 15 mm. It was then completely excised (Figure 2B). The cystic fluid was aspirated. Postoperative semen analysis revealed no sperm cells. Postoperative recovery was uneventful, and the patient was discharged from the hospital. Pathological examination confirmed the diagnosis of a “left epididymal cyst” (Figures 2C,D).

### Case 3

**Figure 3:**
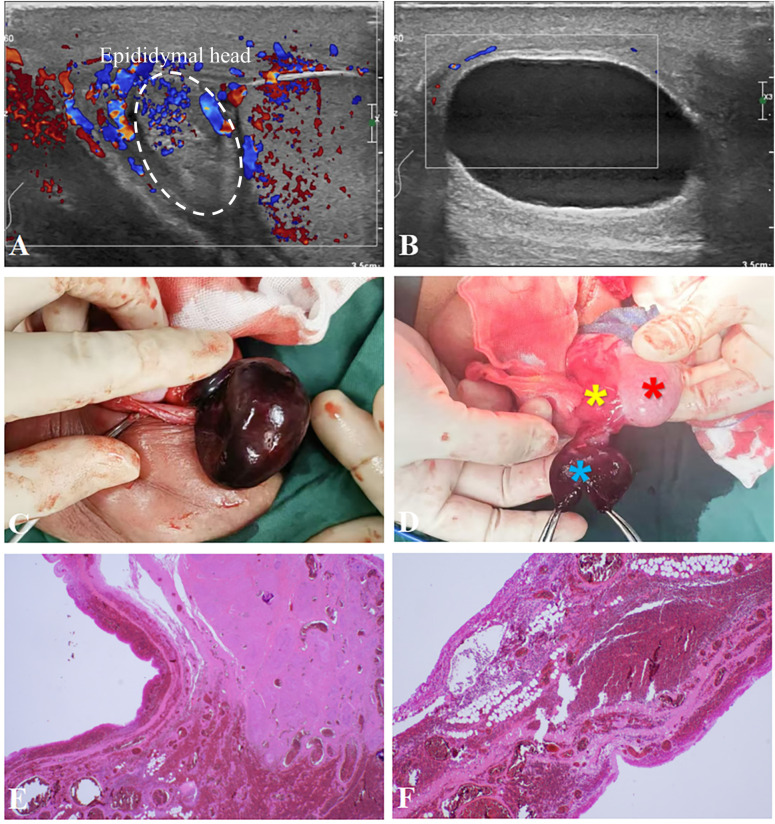
**(A)** scrotal ultrasound revealed enlargement of the right epididymal head, measuring 21 × 19 mm, with reduced internal echogenicity and uneven distribution. Increased internal blood flow was noted, with no obvious mass formation. **(B)** Scrotal ultrasound also showed a cystic mass located between the right epididymis and spermatic cord, with clear boundaries, thick walls, and enhanced echogenicity. No significant blood flow signal was detected within the cyst wall. (According to the sonographer, a ’spiral echo’ was observed at the lower end of the right spermatic cord during the examination; however, it was faint, and the imaging was not conclusive.) **(C)** During surgery, the ischemic cystic mass was found to be purplish-black, approximately 4 × 3 cm in size. **(D)** The cystic mass (blue asterisk) was observed connecting to the epididymal head (yellow asterisk), demonstrating a 720°torsion at the junction. The testis exhibited a 180°counterclockwise torsion, which regained normal coloration (red arrow) after detorsion, transitioning from its initial dusky appearance. **(E,F)** Histopathological examination demonstrated cystic wall tissue partially lined by a single layer of cuboidal to flattened epithelium, with focal areas denuded of epithelial lining. The cyst wall consisted of fibrous connective tissue exhibiting marked vascular congestion, hemorrhage, and focal necrosis.

A 14-year-old adolescent presented to our hospital with a 5-day history of right-sided scrotal pain. Two days prior, the patient had visited our emergency department. On examination, the right scrotum was found to be red and swollen, with tenderness upon palpation, but the Prehn sign was negative. The left side appeared normal. The patient had no prior history of scrotal trauma. Ultrasound examination revealed “enlargement of the right epididymal head measuring 21 × 19 mm, with decreased and uneven internal echogenicity, increased blood flow on Color Doppler Flow Imaging (CDFI), no significant mass” (Figure 3A). The emergency physician diagnosed “right epididymitis.” Treatment with intravenous ceftriaxone for infection and dexamethasone for inflammation was initiated. Two days later, follow-up showed mild improvement in scrotal pain. A repeat scrotal ultrasound was then performed. The examination utilized a Philips EPIQ5 system with a 12 MHz linear transducer, with pulse repetition frequency set at 0.8 kHz and wall filter at 50 Hz to optimize sensitivity for low flow. It demonstrated “a spiral-shaped echo at the lower end of the right spermatic cord; a cystic mass between the right epididymis and spermatic cord with clear borders, thick walls, and enhanced echogenicity; no significant blood flow signals were observed in the cyst wall on CDFI (Figure 3B).” The findings were suggestive of “right testicular torsion; right scrotal cystic mass (possibly epididymal cyst with torsion, spermatic cord cyst to be excluded)."An urgent right scrotal exploration was performed. Intraoperatively, a twisted mass was noted in the right scrotum, measuring approximately 4 × 3 cm, with a purple-black color. The mass was connected to the head of the epididymis and had twisted 720° counterclockwise, with the testicle also twisted 180° counterclockwise. The testicle retained a reddish hue, and both the mass and testicle were detorsed (Figures 3C,D). Following detorsion, the testis exhibited a improved reddish hue. The tunica albuginea was then incised longitudinally to the depth of the medulla to assess blood supply. Immediate active bleeding from the cut edges was observed, confirming satisfactory reperfusion of the testicular parenchyma. Following this, a small incision was made in the mass, and transparent white fluid was expelled. The mass was completely excised. After the mass excision, bilateral orchidopexy was performed. Histopathological examination showed an “epididymal cyst with congestion, hemorrhage, and degenerative necrosis” (Figures 3E,F). The patient recovered and was discharged. Two-week postoperative color Doppler ultrasound confirmed normal blood flow in both testes. The right testis measured 51 × 22 mm and showed heterogeneous echotexture, while the left testis (50 × 20 mm) was normal.

## Discussion

Epididymal cysts (EC) are fluid-filled cystic masses located in the epididymis (6). The exact etiology remains unclear, although some studies suggest a potential link to hormonal imbalances during embryonic development, positioning epididymal cysts as a congenital anomaly (2, 7). Furthermore, maternal intake of hormonal disruptors such as diethylstilbestrol during pregnancy has been associated with an increased risk of fetal epididymal cysts (8). Environmental endocrine disruptors and obstruction of the vas deferens or epididymal duct may also contribute to the formation of epididymal cysts in children (2, 9). Some literature even suggests that epididymal cysts might be a manifestation of testicular hypoplasia syndrome (10–12). Interestingly, patients with epididymal cysts often present with a larger testicular volume on the affected side compared to individuals without such cysts (2, 13).

In children, the incidence of epididymal cysts is reported to range from 5% to 20%, with an increasing prevalence as age advances (2). Approximately 20%–30% of epididymal cysts are detected incidentally during routine physical examinations and confirmed by scrotal ultrasound. Smaller cysts may be asymptomatic and challenging to detect, with up to 70%–80% of cases remaining unnoticed (6, 13, 14). For instance, case 1 and case 2 presented in this report had no significant clinical symptoms. While epididymal cysts and spermatoceles are often considered the same disease in many studies, there are notable differences (6, 15). An epididymal cyst is a broad term referring to cysts typically seen in prepubertal individuals. These cysts are located anywhere within the epididymis and contain serous fluid without sperm. On the other hand, spermatoceles are more commonly seen post-puberty, typically located in the head of the epididymis. The fluid within spermatocele is more viscous, containing non-motile sperm and cellular debris (15). Spermatoceles are thus considered a specific subtype of epididymal cysts (8, 15, 16). Both types of cysts appear as anechoic or hypoechoic masses on ultrasound, with thin walls and occasional septations (14). Differentiation between the two may be achieved by cyst fluid aspiration to assess the presence of sperm (16).

In most cases, epididymal cysts are asymptomatic, and scrotal pain is minimal or absent. It is reported that 60% of cysts, especially those smaller than 3 cm, may slowly degenerate over time (17). Some studies suggest that conservative management with periodic follow-up may be appropriate for asymptomatic patients without significant growth of the cysts (18). However, cysts may occasionally be confused with hydroceles due to their similar presentation on physical examination, including positive translucency. Ultrasound plays a crucial role in the definitive diagnosis, as it can clearly delineate the size, location, and characteristics of the cyst, including the presence of blood flow signals, septations, and echo strength (1, 9). Epididymal cysts are typically located in the head of epididymis, appearing as round anechoic or hypoechoic areas with smooth walls and posterior acoustic enhancement, and they cannot be separated from the epididymis (7, 14). Hydroceles, in contrast, are typically located above the testis or within the spermatic cord and manifest as fluid collections in the peritesticular space. Although ultrasound can distinguish the two conditions in most cases, in certain instances, the differentiation may not be straightforward, as seen in case 1 where ultrasound could not definitively distinguish between a hydrocele and spermatocele. This was clarified by cyst aspiration.

Various treatment options exist for epididymal cysts, including conservative observation, sclerosing agent injections (e.g., phenol, ethanolamine oleate, tetracycline), cyst fluid aspiration, and surgical intervention (19–23). Small cysts (less than 10 mm) are typically managed conservatively, while larger cysts, or those that cause symptoms, may warrant surgery (7). Cyst fluid aspiration and sclerosing agent injection are minimally invasive alternatives that can be performed under local anesthesia in the outpatient setting. These options are less expensive and technically simpler than surgery but may require multiple treatments to achieve satisfactory results (24). Surgical intervention is generally recommended for large, symptomatic cysts, with the goal of complete cyst removal (7, 18), as in our case 2. However, surgery must be performed with meticulous care, particularly in separating the cyst from the epididymis to avoid damage to the epididymis and its blood supply. Surgical removal of the cyst is generally not recommended for patients with fertility concerns due to the risk of damaging the epididymis or causing scar tissue that may lead to fertility issues, particularly in cases involving bilateral cysts (7, 25). In our case 1, cyst aspiration was performed first, with the decision to proceed to surgery reserved for follow-up based on the cyst's behavior. Postoperative complications may include scrotal edema, hematoma, abscess formation, wound infection, cyst recurrence, and epididymal tubule obstruction, though the prognosis for most patients is favorable (7).

Epididymal cyst torsion, while rare, can lead to acute scrotal pain and redness. The diagnosis of torsion should be differentiated from other conditions, including testicular torsion, appendage torsion, spermatic cord torsion, epididymitis, orchitis, scrotal trauma, and testicular tumors (5). In cases of acute scrotal pain, the most critical differential diagnosis is testicular torsion, particularly within the first 6 h of symptom onset, as early intervention is key to preventing testicular injury (17). Ultrasound findings of epididymal cyst torsion often show a lack of blood flow, whereas conditions like epididymitis typically exhibit increased blood flow (4). Unfortunately, no clinical marker currently exists to definitively distinguish epididymal cyst torsion from other scrotal emergencies, and aggressive exploration may be the only reliable method of diagnosis. It has also been suggested that the presence of an epididymal cyst can alter the anatomical position and axis of the ipsilateral testis, thereby increasing the risk of testicular torsion in affected individuals (5). Among the 14 previously reported cases of epididymal cyst torsion (Table 1), none were associated with concomitant ipsilateral testicular torsion. Notably, our Case 3 represents a unique clinical scenario where both the cyst (720°) and testis (180°) underwent torsion—the first documented case of synchronous torsion involving an epididymal cyst and testis.

**Table 1:** Summary of adolescent cases with torsioned epididymal cysts.

Case no.	Year	Age (year)	Author	Side	EC torsion degree	History of scrotal trauma	Ultrasound characteristics	Testicular torsion	Management	Outcome/Follow-up
1st	13	1990	Kaye et al. (26)	Left	360°	No	3 × 5.5 cm EC	No	Exploration	Successful excision, lost to follow-up
2nd	1	1997	Liolios et al. (27)	Left	360°	No	EC was not found	No	Exploration	Successful excision, lost to follow-up
3rd	13	2004	Yılmaz et al. (28)	Left	720°	No	3.8 × 3.5 × 3 cm EC	No	Exploration	Successful excision, lost to follow-up
4th	11	2013	Erikçi et al. (1)	Left	720°	Yes	0.4 cm EC	No	Conservative treatment followed by exploration	Successful excision, no recurrence at 1 year
5th	14	2014	Akın et al. (25)	Bilateral	Unknown	No	Left EC:0.9 × 0.7 cm; Right EC:0.45 × 0.3 cm	No	Exploration	Successful excision, lost to follow-up
6th	14	2015	Ameli et al. (29)	Left	720°	Yes	EC was found	No	Exploration	Successful excision, no recurrence at 3 months
7th	16	2018	Bleve et al. (30)	Right	360°	No	4 × 5 cm EC	No	Exploration	Successful excision, no recurrence at 1 year
8th	13	2019	Messina et al. (17)	Left	Unknown	Yes	3.15 × 1.15 cm EC	No	Conservative treatment followed by exploration	Successful excision, no recurrence at 1 month
9th	8	2020	Ozaal et al. (31)	Right	540°	Yes	4.1 × 1.7 cm EC	No	Conservative treatment followed by exploration	Successful excision, lost to follow-up
10th	4	2022	Kumar et al. (32)	Right	540°	No	3.1 × 1.8 cm EC	No	Exploration	Successful excision, lost to follow-up
11th	11	2023	Vafadar et al. (33)	Right	Unknown	No	3 × 2.3 × 1.8 cm EC	No	Exploration	Successful excision, lost to follow-up
12th	3	2023	Wang et al. (34)	Right	Unknown	No	2.5 × 2.1 cm EC	No	Conservative treatment followed by exploration	Successful excision, no recurrence at 1 year
13th	12	2025	Zhanghuang et al. (35)	Left	360°	No	4.1 × 2.7 × 1.7 cm EC	No	Exploration	Successful excision, no recurrence at 3 months
This case 3	14	2025		Right	720°	No	2.1 × 1.9 cm EC	Yes	Exploration	Successful excision, no recurrence at 2 weeks

**Figure 4:**
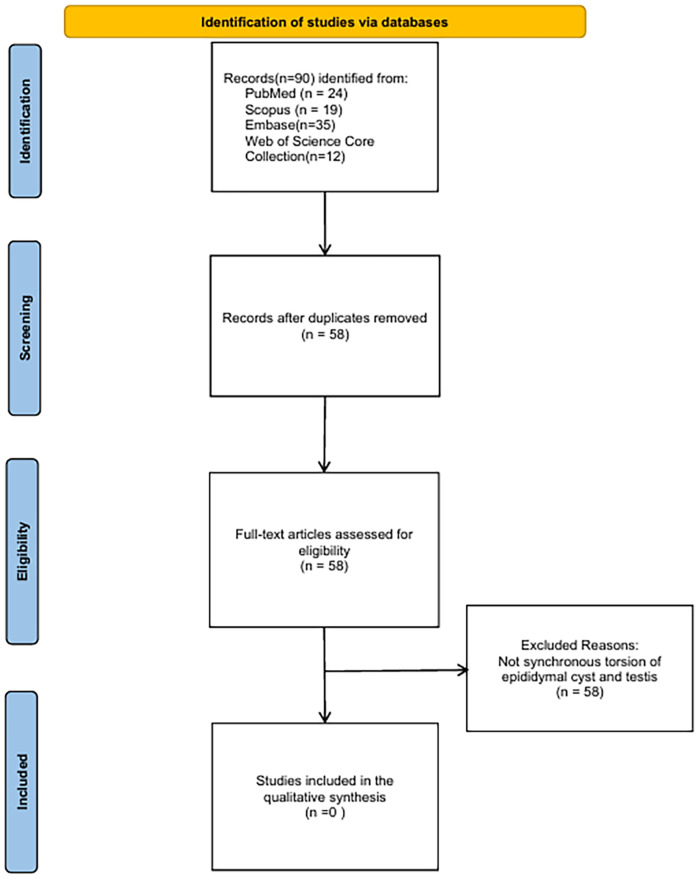
Literature search flow diagram.

To validate the novelty of our case, we performed a systematic literature review up to May 31, 2025, across PubMed, Scopus, Embase, and Web of Science, using the search string: (“epididymal cyst” OR “spermatocele”) AND (“testicular torsion” OR “spermatic cord torsion”). After screening 58 unique publications following deduplication, none of the articles described the synchronous torsion of an epididymal cyst and ipsilateral testis (see PRISMA flow diagram, Figure 4). Therefore, to our knowledge, our Case 3 represents the first documented instance of this unique clinical scenario, where both the cyst (720°) and testis (180°) underwent synchronous torsion.

This finding provides partial validation of our earlier hypothesis while simultaneously suggesting that epididymal cysts may pose greater risks than previously recognized, particularly when large. Patients with long-standing sizable epididymal cysts may experience intermittent discomfort, potentially delaying medical attention. Therefore, patient education should emphasize: (1) recognizing new-onset scrotal pain or swelling, (2) understanding the risk of testicular torsion, and (3) seeking immediate evaluation to prevent diagnostic delays or errors.

Given these complexities, clinicians managing epididymal cysts must be vigilant for the possibility of torsion and promptly perform scrotal exploration to prevent or minimize testicular damage.

A limitation in the management of our case 3 was the absence of immediate microscopic analysis of the aspirated transparent white fluid for the presence of sperm cells. In the acute setting, where the primary clinical concern was the viability of the torsed testis, the surgical team's focus was directed toward rapid exploration and detorsion, and the differentiation between an epididymal cyst and a spermatocele was overlooked. The fluid sample was therefore sent for routine histopathological examination instead of semen analysis. Consequently, a definitive intraoperative differentiation between a simple epididymal cyst and a spermatocele based on fluid composition was not possible. This highlights a valuable learning point: in cases of synchronous pathology where testicular torsion is suspected, a systematic protocol for sampling and analyzing cystic fluid, even amidst surgical urgency, could provide crucial diagnostic information and enhance postoperative counseling.

## Conclusion

Adolescent epididymal cysts are common benign lesions that are often mistaken for hydroceles. A spermatocele, which is a specific subtype of epididymal cyst, contains semen. Aspiration or elective excision are effective for non-acute cases. However, the cyst may undergo torsion and potentially induce testicular torsion, representing a previously underrecognized clinical risk. Given the unique characteristics of this developmental stage, it is crucial to provide personalized treatment strategies for adolescents with epididymal cysts.

## Data Availability

The original contributions presented in the study are included in the article/Supplementary Material, further inquiries can be directed to the corresponding authors.

## References

[1] Erikçi V Hoşgör M Yıldız M Aksoy N Okur Ö Örnek Y Torsion of an epididymal cyst: a case report and review of the literature. Turk J Pediatr. (2013) 55(6):659–61. 24577990

[2] Erikci V Hoşgör M Aksoy N Okur Ö Yildiz M Dursun A Management of epididymal cysts in childhood. J Pediatr Surg. (2013) 48(10):2153–6. 10.1016/j.jpedsurg.2013.01.058 24094972

[3] Arora B Arora R Arora A. A randomised study of management modalities in epididymal cyst. Int Surg J. (2019) 6:340–4. 10.18203/2349-2902.isj20190379

[4] Ilangovan GMM Mounika V Khan MA. Torsion of epididymal cyst: a case report with review of literature. Cureus. (2023) 15(12):e51158. 10.7759/cureus.51158 38283501 PMC10811970

[5] Gebreselassie KH Berhanu E Akkasa SS Woldehawariat BY. Torsed spermatocele, a rare cause of acute scrotum: report of a case and review of literature. Urol Case Rep. (2022) 45:102172. 10.1016/j.eucr.2022.102172 35959225 PMC9357836

[6] Homayoon K Suhre CD Steinhardt GF. Epididymal cysts in children: natural history. J Urol. (2004) 171(3):1274–6. 10.1097/01.ju.0000110322.87053.99 14767330

[7] Boscarelli A Bellini T. Epididymal cyst in children. Eur J Pediatr. (2021) 180(9):2723–9. 10.1007/s00431-021-04080-5 33851241

[8] Chung SE Frush DP Fordham LA. Sonographic appearances of extratesticular fluid and fluid-containing scrotal masses in infants and children: clues to diagnosis. AJR Am J Roentgenol. (1999) 173(3):741–5. 10.2214/ajr.173.3.10470915 10470915

[9] Woodward PJ Schwab CM Sesterhenn IA. From the archives of the AFIP: extratesticular scrotal masses: radiologic-pathologic correlation. Radiographics. (2003) 23(1):215–40. 10.1148/rg.231025133 12533657

[10] Skakkebaek NE Rajpert-De Meyts E Main KM. Testicular dysgenesis syndrome: an increasingly common developmental disorder with environmental aspects. Hum Reprod. (2001) 16(5):972–8. 10.1093/humrep/16.5.972 11331648

[11] Sharpe RM. Hormones and testis development and the possible adverse effects of environmental chemicals. Toxicol Lett. (2001) 120(1-3):221–32. 10.1016/s0378-4274(01)00298-3 11323180

[12] Jensen TK Toppari J Keiding N Skakkebaek NE. Do environmental estrogens contribute to the decline in male reproductive health? Clin Chem. (1995) 41(12 Pt 2):1896–901. 10.1093/clinchem/41.12.1896 7497651

[13] Posey ZQ Ahn HJ Junewick J Chen JJ Steinhardt GF. Rate and associations of epididymal cysts on pediatric scrotal ultrasound. J Urol. (2010) 184(4 Suppl):1739–42. 10.1016/j.juro.2010.03.118 20728143

[14] O'Kelly F McAlpine K Abdeen N Keays MA Guerra LA Leonard MP. The futility of continued surveillance of epididymal cysts - a study of the prevalence and clinico-demographics in pre- vs. post-pubertal boys. Can Urol Assoc J. (2019) 13(12):E398–e403. 10.5489/cuaj.5667 31039113 PMC6892700

[15] Weatherly D Wise PG Mendoca S Loeb A Cheng Y Chen JJ Epididymal cysts: are they associated with infertility? Am J Mens Health. (2018) 12(3):612–6. 10.1177/1557988316644976 27118455 PMC5987960

[16] Salama N Hassan OS. A post-aspiration giant spermatocele in a young man: a case report and literature review. Clin Med Insights Case Rep. (2022) 15:11795476221097218. 10.1177/11795476221097218 35591974 PMC9112308

[17] Messina M Fusi G Ferrara F Bindi E Pellegrino C Molinaro F A rare cause of acute scrotum in a child: torsion of an epididymal cyst. Case report and review of the literature. Pediatr Med Chir. (2019) 41(1):210. 10.4081/pmc.2019.210 31232012

[18] Cai W Liu C Xu L Wu Q Kuang T Lin X. Epididymal cysts in children: frequency, clinical characteristics, and management strategies. Front Pediatr. (2024) 12:1455866. 10.3389/fped.2024.1455866 39108693 PMC11300225

[19] Ahmed M. Sclerotherapy with aqueous phenol for outpatient treatment of hydrocoele and epididymal cysts. J Pak Med Assoc. (1992) 42(7):159–60. 1404833

[20] Bullock N Thurston AV. Tetracycline sclerotherapy for hydroceles and epididymal cysts. Br J Urol. (1987) 59(4):340–2. 10.1111/j.1464-410x.1987.tb04645.x 3580774

[21] Hellström P Malinen L Kontturi M. Sclerotherapy for hydroceles and epididymal cysts with ethanolamine oleate. Ann Chir Gynaecol. (1986) 75(1):51–4. 3518591

[22] Nash JR. Sclerosant treatment for hydroceles and epididymal cysts. Br Med J. (1980) 280(6208):182–3. 10.1136/bmj.280.6208.182-b 7357315 PMC1600345

[23] Moloney GE. Comparison of results of treatment of hydrocele and epididymal cysts by surgery and injection. Br Med J. (1975) 3(5981):478–9. 10.1136/bmj.3.5981.478 1156828 PMC1674287

[24] Low LS Nair SM Davies AJW Akapita T Holmes MA. Aspiration and sclerotherapy of hydroceles and spermatoceles/epididymal cysts with 100% alcohol. ANZ J Surg. (2020) 90(1-2):57–61. 10.1111/ans.15467 31628703

[25] Akin Y Sarac M Basara I Yucel S Kazez A. Bilateral epididymal cyst in 14 year-old boy: a case report. Journal of Health Sciences. (2014) 4(1):68–71. 10.17532/jhsci.2014.150

[26] Kaye RI Cromie WJ. Torsion of a spermatocele: a case report and review of the literature. J Urol. (1990) 143(4):786. 10.1016/s0022-5347(17)40094-2 2313807

[27] Liolios N Anagnostopoulos D Sinopidis X Vassou N Kasselas V. Torsion of spermatocele and aplasia of the vas deferens. A case report. Eur J Pediatr Surg. (1997) 7(2):118–9. 10.1055/s-2008-1071069 9165262

[28] Yilmaz E Batislam E Bozdogan O Basar H Basar MM. Torsion of an epididymal cyst. Int J Urol. (2004) 11(3):182–3. 10.1111/j.1442-2042.2003.00764.x 15009369

[29] Ameli M Boroumand-Noughabi S Gholami-Mahtaj L. A 14-year-old boy with torsion of the epididymal cyst. Case Rep Urol. (2015) 2015:731987. 10.1155/2015/731987 26798545 PMC4698546

[30] Bleve C Conighi ML Bucci V Costa L Chiarenza SF. Torsion of huge epididymal cyst in a 16-year-old boy: case report and review of the literature. Pediatr Med Chir. (2018) 40(1):162. 10.4081/pmc.2018.162 29871476

[31] Ozaal A Pragalathan B Lavanya S Sarma ST. Torsion of an epididymal cyst: a rare finding on scrotal exploration for acute scrotum. Case Rep Urol. (2020) 2020:8858606. 10.1155/2020/8858606 33299634 PMC7704176

[32] Kumar R Kataria R Singh H Jassal A. Rare case of epidydimal cyst torsion causing acute scrotum in 4 yr old boy. Int J Med Rev Case Rep. (2022) 6(13):29.

[33] Vafadar M Rakhshankhah N Salek M Zarei E. Torsion of epididymal cyst as a cause of acute scrotum in a child. Urol Case Rep. (2023) 48:102417. 10.1016/j.eucr.2023.102417 37215055 PMC10192393

[34] Wang Y Wang K Yu L Ma X. A rare case of scrotal emergency: torsion of epididymal cyst-a case report and literature review. Front Pediatr. (2023) 11:1245842. 10.3389/fped.2023.1245842 38264504 PMC10803493

[35] Zhanghuang C Li J Long N Duan Q Ji F Xie Y Torsion of a giant epididymal cyst in a pediatric patient: a rare cause of acute scrotal pain-case report and literature review. Front Pediatr. (2025) 13:1605254. 10.3389/fped.2025.1605254 40492265 PMC12146361

